# A Cross-Platform Comparison of Genome-Wide Expression Changes of Laser Microdissected Lung Tissue of C-Raf Transgenic Mice Using 3′IVT and Exon Array

**DOI:** 10.1371/journal.pone.0040778

**Published:** 2012-07-16

**Authors:** Kishor Bapu Londhe, Juergen Borlak

**Affiliations:** Centre for Pharmacology and Toxicology, Hannover Medical School, Hannover, Germany; National Cancer Center, Japan

## Abstract

Microarrays are widely used to study genome-wide gene expression changes in different conditions most notably disease, growth, or to investigate the effects of drugs on entire genomes. While the number and gene probe sequences to investigate individual gene expression changes differs amongst manufactures, the design for all of the probes is biased towards the 3′ region. With the advent of exon arrays, transcripts of any known or predicted exon can be investigated to facilitate the study of genome-wide alternative splicing events. Thus, the use of exon arrays provides unprecedented opportunities in gene expression studies. However, it remains a major challenge to directly compare gene expression data derived from oligonucleotide to exon arrays. In the present study, genome-wide expression profiling of Laser Micro-dissected Pressure Catapulted (LMPC) samples of *c-Raf* mouse lung adenocarcinoma, dysplasia, unaltered transgenic and non-transgenic tissues was performed using the Affymetrix GeneChip Mouse Genome 430 2.0 Array and whole genome Mouse Exon 1.0 ST Array. Based on individual group comparisons 52 to 83% of regulated genes were similar in direction, but fold changes of regulated genes disagreed when data amongst the two platforms were compared. Furthermore, for 27 regulated genes opposite direction of gene expression was observed when the two platforms were compared pointing to the need to assess alternative splicing events at the 3′ end. Taken collectively, exon arrays can be performed even with laser microdissected samples but fold change gene expression changes differ considerably between 3′IVT array and exon arrays with alternative splicing events contributing to apparent differences in gene expression changes.

## Introduction

Microarrays are promising tools to identify genetic changes due to different diseases conditions and several manufactures provide platform solutions; however the data obtained can be affected by technical, instrumental, computational factors and data interpretation. Moreover, the reproducibility and accuracy of microarray data has been the subject of independent reports [1], [2], [3], [4], [5]. The MicroArray Quality Control (MAQC) project had compared various microarray platforms to define reproducibility of data between different platforms [6]. A further addition to the study of whole genomes is the Affymetrix Whole Transcript Exon Sense Target array (Exon 1.0 ST) to enable the detailed analysis of transcripts at the exon expression level thereby facilitating investigations into alternative splicing events. The Affymetrix exon array is an impressive dense array featuring more than 1.2 million probesets for the study of any known or predicted exon in the genome. The exon array is therefore a powerful tool to study alternative splicing events that might occur in various stages of cell life alteration like disease, growth and differentiation [7], [8].

**Figure 1:**
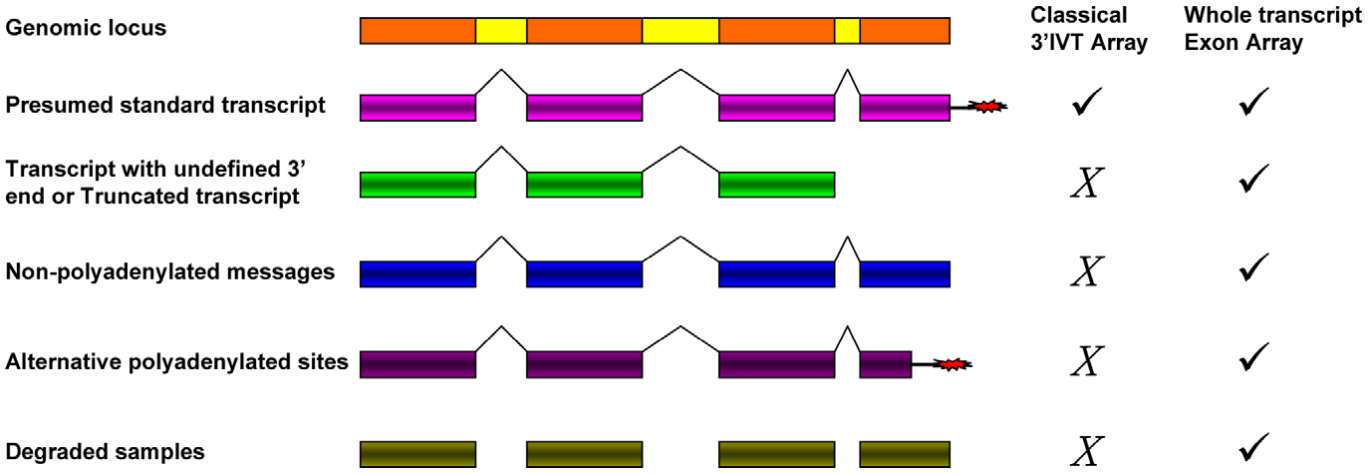
Probe selection region in exon array and 3′IVT array. Exon array has at least 1 probeset per exon and there might be 2–4 probes per probeset. Probesets in exon array cover over whole transcript region. In 3′IVT array the probeset is at 3′ end of transcript. Adapted from (http://media.affymetrix.com/support/technical/datasheets/exon_arraydesign_datasheet.pdf).

**Figure 2:**
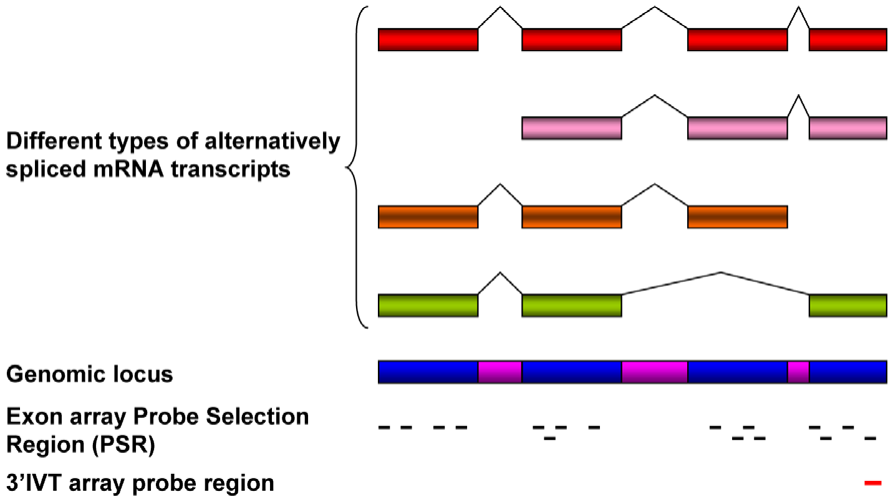
Types of transcripts detected by Whole Transcript Exon Array. Whole Transcript Exon Array has ability to detect transcripts even in degraded samples and transcripts missing or alternative polyadenylated tail. Classical 3′IVT array lack ability to detect different transcript variations. Adapted from (http://media.affymetrix.com/support/technical/appnotes/wt_ appnote.pdf).

Conventionally, scientists use 3′IVT expression array which informs on the total gene expression changes only [9]. However, the strategy for probe design in the exon array differs from that of the 3′IVT expression array. With the exon array the probes are designed to detect specific exons in the transcripts while for the 3′IVT array 11 probe pairs of perfect match and corresponding mismatch probes are designed towards the 3′ end to detect the transcripts With exon arrays there are 26 bins of background probes having varying GC count for the entire array but not against each transcript as with the 3′IVT array [8]. The signals above the background level are only calculated. Each probe score is corrected for background by subtracting the median expression score of background probes of similar GC content from the same chip. With exon array there is at least one probeset comprising 1 to 4 probes against an exon (Figure 1). Furthermore, the exon array has 3 kinds of probesets namely the core, extended and full probesets to determine exon specific transcript expression. The core probes consists of alignments of mRNA with annotated full-length coding DNA sequence (CDS) regions, the extended type consists of all annotations based on cDNA alignments, while the full type consisting of sets of ab-initio gene predictions additionally to all annotations based on cDNA [8], [10]. Exon arrays employ T7 linked random hexamers as compared to T7 oligo-dT primers for cDNA synthesis used in 3′IVT array. T7 oligo-dT primers require intact poly-A tail; therefore, some genes may not be accurately represented on a 3′IVT expression array in situations like truncated transcript, loss of poly-A tail and degraded samples (Figure 2) [7]. Unlike 3′IVT, arrays exon arrays require whole transcript sense target labeling and it generates single strand sense DNA for DNA/DNA hybridization. Previous studies investigated the signal intensity correlation between 3′IVT array and exon array and suggest that the probe signal intensity obtained by exon array is less than that of 3′IVT array probes [11]. One study with bronchial brushing samples from lung cancer patients reported better and more reproducible results with exon as compared to 3′IVT arrays [12] and Gardina et al., 2007 reported a good correlation between paired Affymetrix human exon array and 3′IVT array using breast tissue [13].

**Table 1:** Comparison between 3′IVT microarray with FDR<0.001 and exon array gene expression fold change levels.

Comparison groups	No. of total differentiallyexpressed genesidentified with 3′IVT array	No. of common differentially expressed genes found in the comparison exonarray versus 3′IVT array (%)		
		Full probe level	Extended probe level	Core probe level
Dysplasia vs Transgenic	95	68 (71.58%)	68 (71.58%)	63 (66.32%)
Dysplasia vs Non-transgenic	237	157 (66.24%)	155 (65.40%)	129 (54.43%)
Tumor vs Transgenic	359	283 (78.83%)	275 (76.60%)	239 (66.57%)
Tumor vs Non-transgenic	404	337 (83.42%)	332 (82.18%)	292 (72.28%)

Number of common genes found in comparison between 3′IVT and exon array. For 3′IVT array differential gene expression fold change obtained using ArrayTrack software with parameters as false detection rate (FDR) <0.001, fold change of ≥2, mean channel intensity (MCI) of 100, bad flag of 5. For exon array differential gene expression fold change obtained using Biotique XRAY software at p<0.05 using a minimum probeset per gene of 2 and a minimum of 2 probes per probeset. In the () are % of genes found common in exon array from 3′IVT array.

**Table 2:** Comparison between 3′IVT microarray with FDR<0.05 and exon array gene expression fold change levels.

Comparison groups	No. of total differentiallyexpressed genesidentified with 3′IVT array	No. of common differentially expressed genes found in the comparison exonarray versus 3′IVT array (%)		
		Full probe level	Extended probe level	Core probe level
Dysplasia vs Transgenic	1536	895 (58.27%)	867 (56.45%)	801 (52.15%)
Dysplasia vs Non-transgenic	2147	1362 (63.44%)	1353 (63.02%)	1165 (54.26%)
Tumor vs Transgenic	1628	1087 (66.77%)	1058 (64.99%)	931 (57.19%)
Tumor vs non-transgenic	2363	1672 (70.76%)	1651 (69.87%)	1455 (61.58%)

Number of common genes found in comparison between 3′IVT and exon array. For 3′IVT array differential gene expression fold change obtained using ArrayTrack software with parameters as false detection rate (FDR) of <0.05, fold change ≥2, mean channel intensity (MCI) of 100, bad flag of 5. For exon array differential gene expression fold change obtained using Biotique XRAY software at p<0.05 using a minimum probeset per gene of 2 and a minimum of 2 probes per probeset. In the () are % of genes found common in exon array from 3′IVT array.

**Figure 3:**
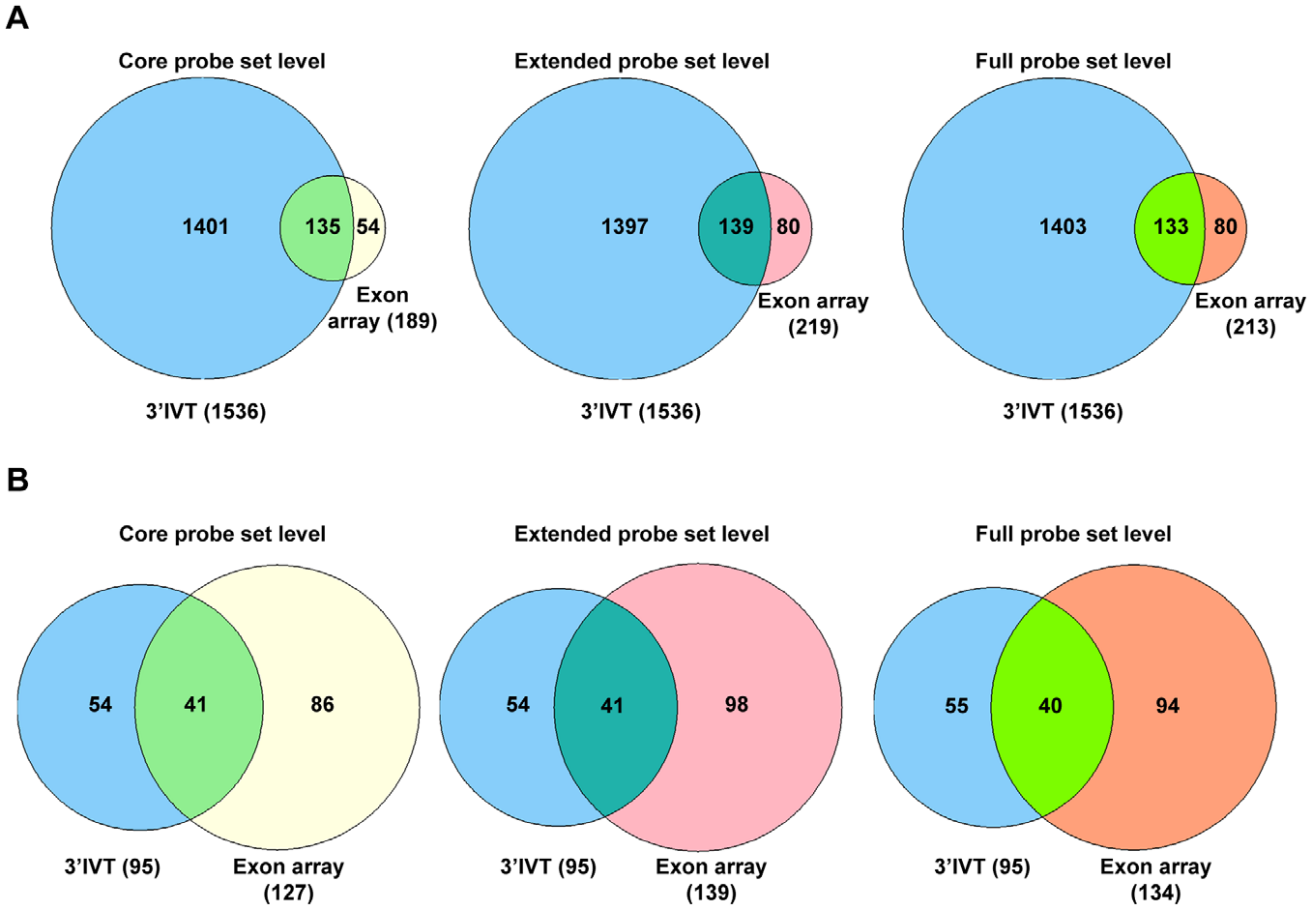
Gene number comparison 3′IVT array versus exon array for dysplasia versus transgenic lung tissue at core, extended and full probeset levels. A. 3′IVT array analyzed with ArrayTrack software at FDR of 0.05, fold change ≥2, mean channel intensity = 100, bad flag = 5, and exon array analyzed with Biotique XRAY software at p<0.05, fold change ≥2, minimum probesets per genes = 2, minimum probes per probeset = 2. B. 3′IVT array analyzed with ArrayTrack software at FDR of 0.001, Fold change ≥2 and exon array analyzed with Biotique XRAY software at p<0.001, fold change ≥2, minimum probesets per genes = 2, minimum probes per probeset = 2.

**Figure 4:**
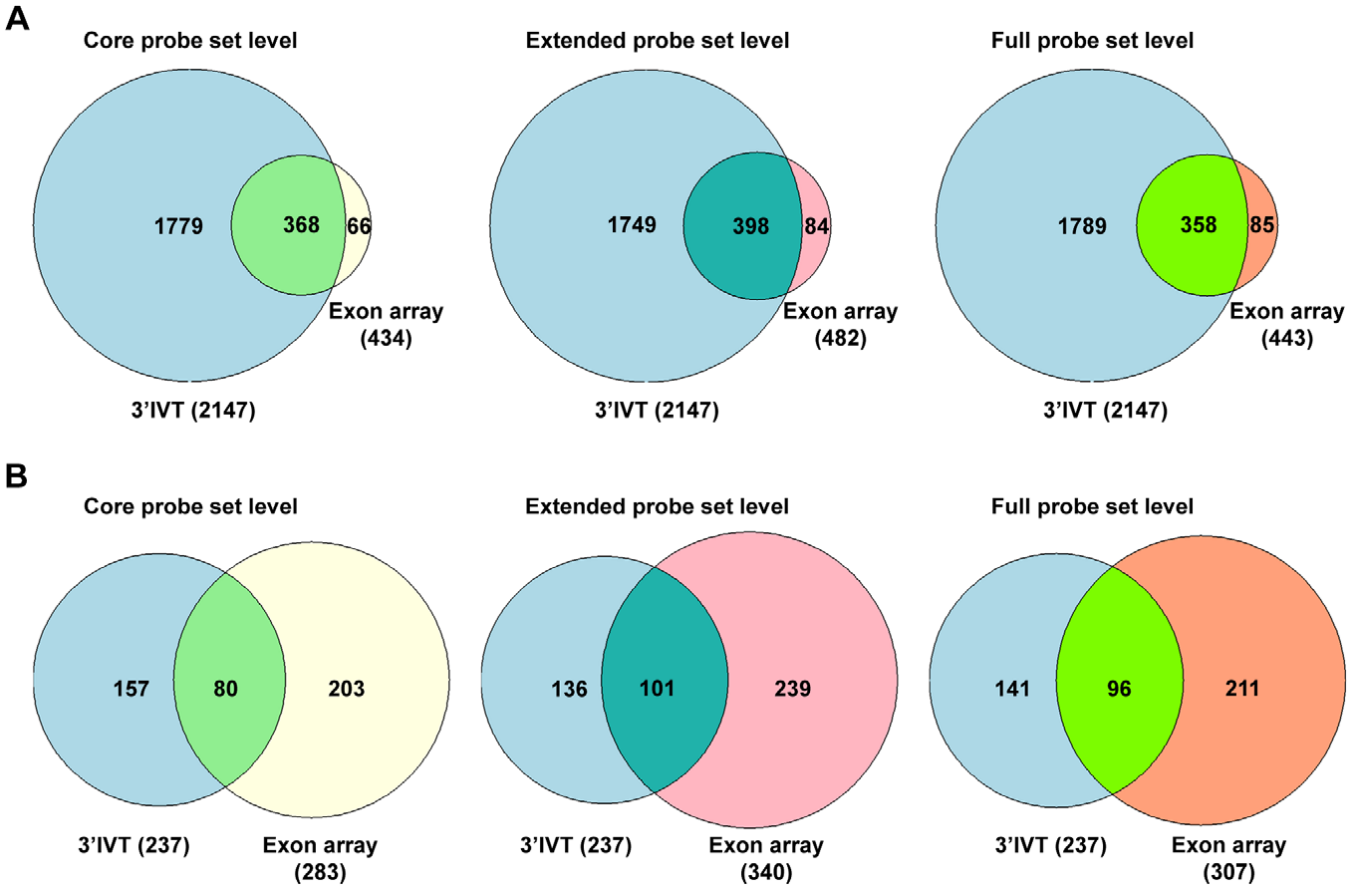
Gene number comparison 3′IVT Array versus exon array for dysplasia versus non-transgenic lung tissue at core, extended and full probeset levels. A. 3′IVT array analyzed with ArrayTrack software at FDR of 0.05, fold change ≥2, mean channel intensity = 100, bad flag = 5, and exon array analyzed with Biotique XRAY software at p<0.05, fold change ≥2, minimum probesets per genes = 2, minimum probes per probeset = 2. B. 3′IVT array analyzed with ArrayTrack software at FDR of 0.001, Fold change ≥2 and exon array analyzed with Biotique XRAY software at p<0.001, fold change ≥2, minimum probesets per genes = 2, minimum probes per probeset = 2.

**Figure 5:**
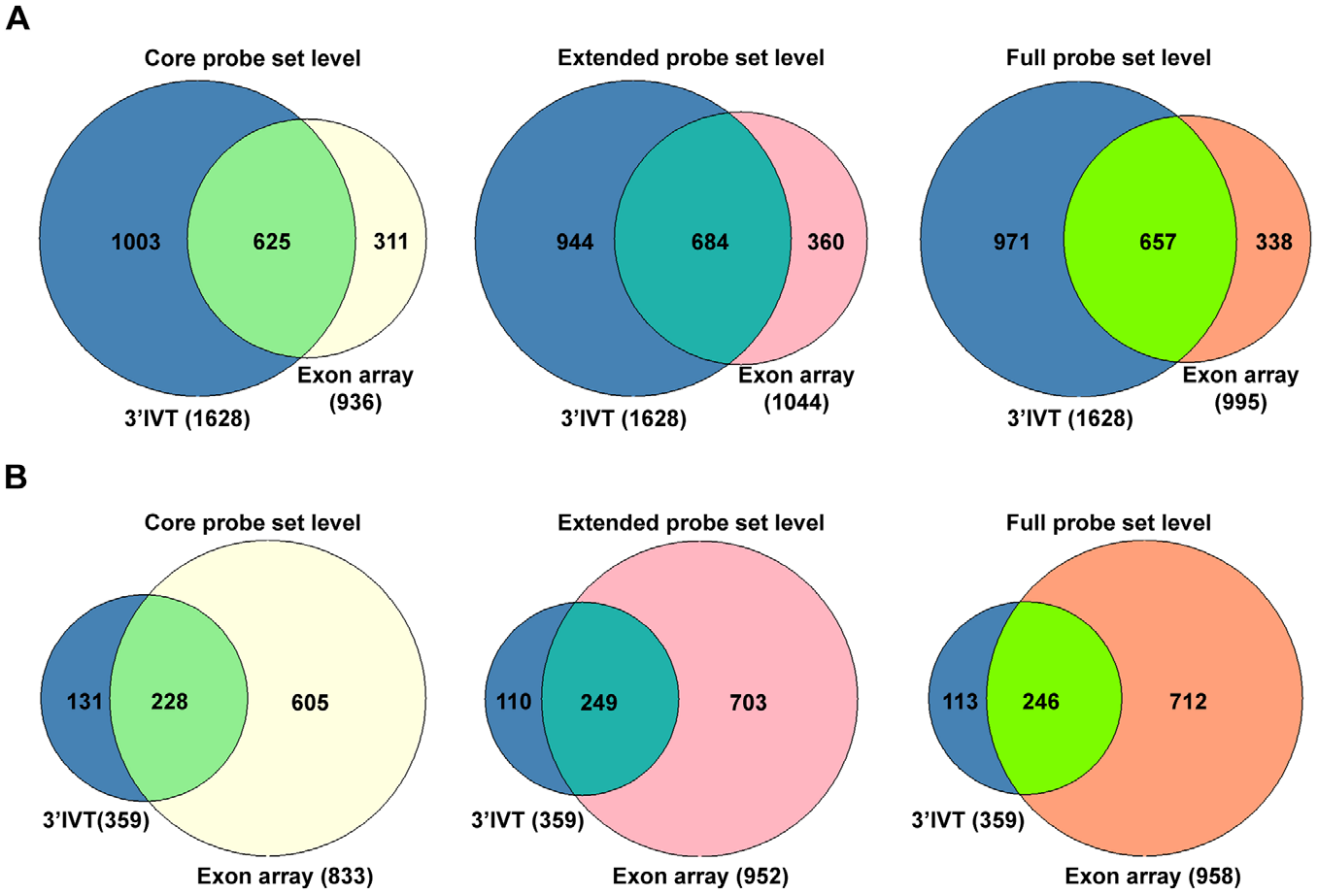
Gene number comparison 3′IVT array versus exon array for tumor versus transgenic lung tissue at core, extended and full probeset levels. A. 3′IVT array analyzed with ArrayTrack software at FDR of 0.05, fold change ≥2, mean channel intensity = 100, bad flag = 5, and exon array analyzed with Biotique XRAY software at p<0.05, fold change ≥2, minimum probesets per genes = 2, minimum probes per probeset = 2. B. 3′IVT array analyzed with ArrayTrack software at FDR of 0.001, Fold change ≥2 and exon array analyzed with Biotique XRAY software at p<0.001, fold change ≥2, minimum probesets per genes = 2, minimum probes per probeset = 2.

**Figure 6:**
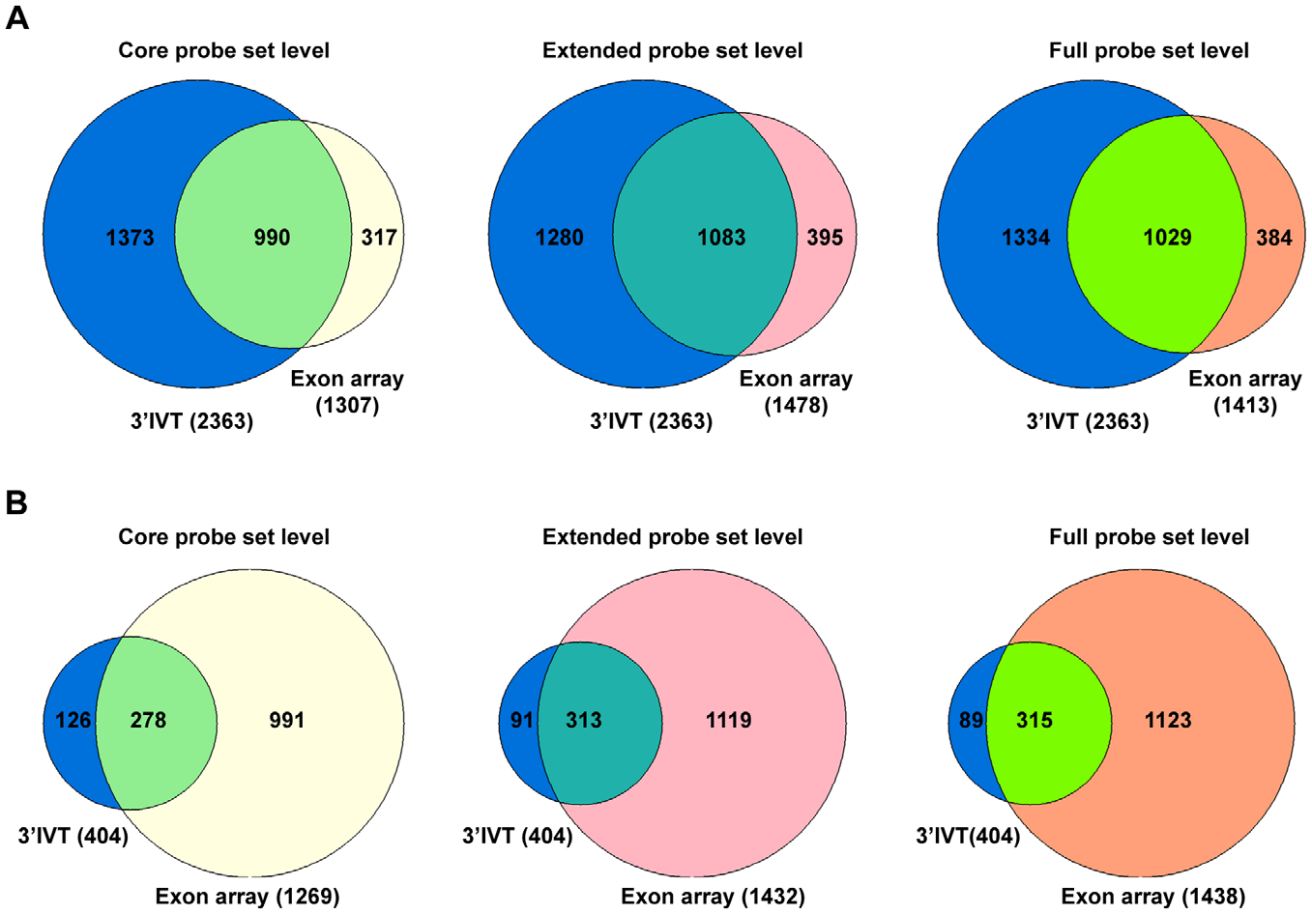
Gene number comparison 3′IVT array versus exon array for tumor versus non-transgenic lung tissue at core, extended and full probeset levels. A. 3′IVT array analyzed with ArrayTrack software at FDR of 0.05, fold change ≥2, mean channel intensity = 100, bad flag = 5, and exon array analyzed with Biotique XRAY software at p<0.05, fold change ≥2, minimum probesets per genes = 2, minimum probes per probeset = 2. B. 3′IVT array analyzed with ArrayTrack software at FDR of 0.001, Fold change ≥2 and exon array analyzed with Biotique XRAY software at p<0.001, fold change ≥2, minimum probesets per genes = 2, minimum probes per probeset = 2.

However, these studies had not considered changes in direction of expression fold changes obtained by use of the exon and 3′IVT array. Okoniewski et al., 2007 mentioned high correlation between Human exon array and 3′IVT array in comparing various tissues but their data showed change in direction of fold change for few genes only [14]. Robinson and Speed, 2007 compared data between Human Genome U113 Plus 2.0 (U133) (3′IVT), Human Gene 1.0 ST (HuGene) and Human Exon 1.0 ST array [15]. They found for exon array the gene-level expressions are less reproducible than the other two arrays, despite having the largest average number of probes per gene. Nonetheless, the correlation between the probeset intensities was around 0.80 for all 3 arrays with some non-linearity due to higher signal intensities from exon arrays. Around 65% differentially expressed genes overlap in all 3 arrays but no data on the correlation at fold change gene expression changes was shown.

**Table 3:** Comparison between 3′IVT microarray with FDR<0.001 and exon array gene expression fold change levels using ArrayTrack platform.

Comparison groups	No. of differentially expressedgenes in exon array	No. of differentially expressedgenes in 3′IVT array	No. of common genes in exonversus 3′IVT array
Dysplasia vs Transgenic	933	95	42 (44.21%)
Dysplasia vs Non-transgenic	1677	237	111 (46.84%)
Tumor vs Transgenic	4319	359	273 (76.04%)
Tumor vs Non-transgenic	4436	404	317 (78.47%)

Number of common genes found when same platform of ArrayTrack software used for comparison between 3′IVT and exon array. For 3′IVT array differential gene expression fold change obtained with parameters as false detection rate (FDR) <0.001, fold change of ≥2, mean channel intensity (MCI) of 100, bad flag of 5. For exon array differential gene expression fold change obtained with parameters as false detection rate (FDR) of <0.05, mean channel intensity (MCI) of 100, bad flag of 4. In the () are % of genes found common in exon array from 3′IVT array.

**Table 4:** Comparison between 3′IVT microarray with FDR<0.05 and exon array gene expression fold change levels using ArrayTrack platform.

Comparison groups	No. of differentially expressedgenes in exon array	No. of differentially expressedgenes in 3′IVT array	No. of common genes in 3′IVTarray versus exon array
Dysplasia vs Transgenic	933	1536	238 (15.49%)
Dysplasia vs Non-transgenic	1677	2147	730 (34.00%)
Tumor vs Transgenic	4319	1628	1050 (66.50%)
Tumor vs Non-transgenic	4436	2363	1512 (63.99%)

Number of common genes found when same platform of ArrayTrack software used for comparison between 3′IVT and exon array. For 3′IVT array differential gene expression fold change obtained with parameters as false detection rate (FDR) <0.05, fold change of ≥2, mean channel intensity (MCI) of 100, bad flag of 5. For exon array differential gene expression fold change obtained with parameters as false detection rate (FDR) of <0.05, mean channel intensity (MCI) of 100, bad flag of 4. In the () are % of genes found common in exon array from 3′IVT array.

We previously reported genome-wide gene expression data for the transgenic *c-Raf* lung adenocarcinoma mouse model and compared different disease conditions, i.e. adenocarcinomas, dysplasia, transgenic unaltered versus non-transgenic lung tissue [16], [17]. Notably, dysplasia is characterized by microscopic pathological changes as anisocytosis (loss of uniformity, unequal sized cells), poikilocytosis (abnormally shaped cells), hyperchromatic and large nucleus (darkly stained nucleus and nucleus to cytoplasm ratio increased), presence of mitotic figures (dividing cells) and basement membrane intact [18]. In low grade dysplasia cells are cuboidal and slightly pleomorphic with medium sized nuclei; mitosis is absent. In high grade dysplasia the cells are large and columnar with cytoplasmic pleomorphism; the nuclei are large hyperchromatic with uneven chromatin structure and mitosis can be clearly seen. In the present study we found dysplasia was a focal lesion often 500 µm or less in diameter with distinct border within otherwise normal alveolar tissue. c-RAF is a serine/threonine protein kinase and a direct downstream effecter of RAS. Over-expression of *c-Raf* in alveolar epithelium is achieved by use of the SP-C promoter which is specifically activated in these cells [19]. By the age of 4–5 months dysplastic changes occur and around 8–10 months adenocarcinomas are visible in lung tissue.

**Figure 7:**
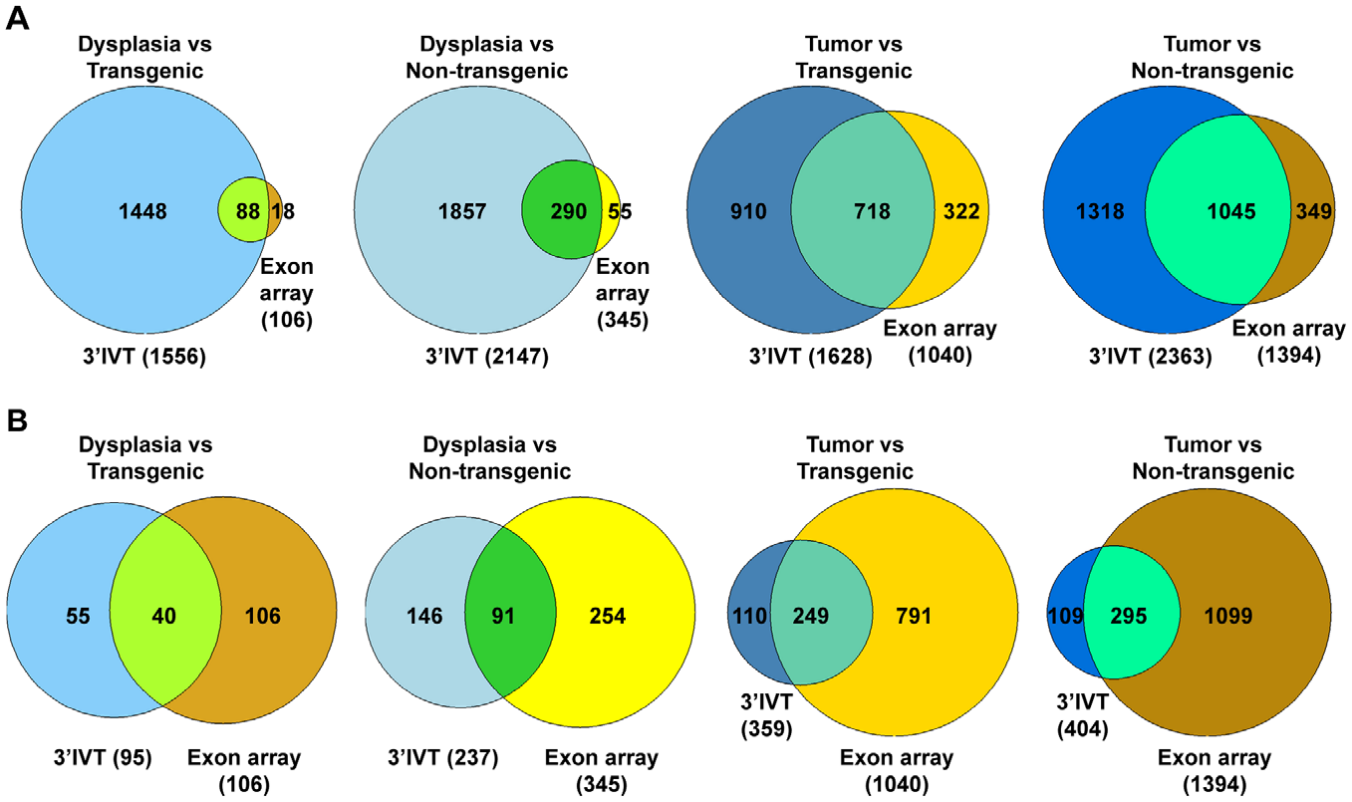
Gene number comparison 3′IVT array versus exon array using ArrayTrack. Comparison between 3′IVT and exon array shows number of genes in common for comparisons Dysplasia vs Transgenic, Dysplasia vs Non-transgenic, Tumor vs Transgenic and Tumor vs Non-transgenic. For exon array gene expression estimated at core probeset level using expression console and analyzed with ArrayTrack software at FDR of 0.05, fold change ≥2, mean channel intensity = 100, bad flag = 4, compared with 3′IVT array analyzed with ArrayTrack software where in **A.** 3′IVT analyzed at criteria at FDR of 0.05, fold change ≥2, mean channel intensity = 100, bad flag = 5. **B.** 3′IVT analyzed criteria at FDR of 0.001, fold change ≥2, mean channel intensity = 100, bad flag = 5.

We aimed for a direct platform comparison of 3′IVT and the exon array by comparing whole genome gene expression data using the same mouse model and the same disease conditions. To this effect we employed the Laser Microdissected Pressure Catapulted (LMPC) method to avoid contamination of surrounding tissue [20], [21], [22], [23].

## Materials and Methods

### Mouse Model

SP-C/*c-Raf* transgenic mice were obtained from the laboratory of Prof. Ulf Rapp (University of Würzburg, Germany), who bred the mice in the C57BL/6/DBA/2 hybrid background. We kept the SP-C/*c-Raf* transgenic mice in the C57BL/6 background for at least five generations. All animal work followed strictly the Public Health Service (PHS) Policy on Human Care and Use of Laboratory Animals. Formal approval to carry out animal studies was granted by the institutional ethics board of the Fraunhofer Institute ITEM, Hannover, and animal procedures were approved by the Lower Saxony State Office for Consumer Protection and Food Safety (Niedersächsisches Landesamt für Verbraucherschutz und Lebensmittelsicherheit, LAVES, Hannover), Germany, reference number 33-42502-06/1081. The samples analyzed in the present study stem from recently conducted work as referenced in [16], [17].

### Tissue Isolation and Laser Micro-dissected Sample Preparation

Lung tissue from transgenic animals aged between 5 months to 12 months and healthy wild mice of age 7 to 10 months were obtained and stored at −80°C until further analysis. Histopathological diagnosis of the lung tissues was done using Haematoxylin and Eosin staining. Using a cryomicrotome slices of 10 µm lung tissue were prepared and fixed over PEN membrane slide (Zeiss GmbH) and stained with Haematoxylin. The desired cells either dysplastic or transgenic (unaltered, morphologically normal) or tumor or wild alveolar cells were laser micro-dissected and collected in an adhesive cap using the LMPC (Laser Micro-dissection Pressure Catapulting) technique. LMPC (Zeiss, P.A.L.M. Microlaser Technologies GmbH) is a method to selectively micro-dissect desired cells from tissue section or live cell monolayer and to collect it in an adhesive cap without contact and contamination [20], [21], [22], [23].

### RNA Isolation and Microarray Hybridization Experiments

RNA extraction was done with the RNeasy Micro Kit (Qiagen, Santa Clarita, CA, USA). The extracted RNA was checked for quality on Agilent 2100 Bioanalyzer (Agilent Technologies, Palo Alto, USA). The RNA samples having RNA integrity number (RIN) above 4 were selected for exon array (Affymetrix).

1 µg of total RNA from individual animals of n = 4 dysplasia/transgenic unaltered paired specimens and lung adenocarcinoma as well as n = 5 non-transgenic lung tissues were used for rRNA reduction and RNA labeling using the Invitrogen and Affymetrix kits, respectively, according to the manufacturer’s instructions. Hybridization cocktails containing 4.5–5 µg of fragmented, end-labeled single-stranded sense target cDNA were prepared and hybridized to GeneChip Mouse Exon 1.0 ST arrays. Processed arrays were scanned using the GeneChip Scanner 3000 7G. Affymetrix Expression Console Software (version 1.0) was used to perform quality assessment.

Five samples each of dysplastic tissue, transgenic but otherwise unaltered tissue, tumor and non-transgenic tissue from SPC/*c-Raf* mice lung were laser micro-dissected and similar methods and quality controls were used to isolate RNA. Mouse 430 2.0 microarray chip from Affymetrix was used to perform 3′IVT array according to manufacturer’s instruction and protocol as per discussed in detail in our previous publications [16], [17].

### Statistical Analysis

Differential gene expression for exon array was analyzed using two different softwares, ArrayTrack (v 3.4.5) and Biotique XRAY (v 3.2). The following criteria were used for exon array differential gene expression study using the Biotique XRAY software.

The mixed model nested ANOVA method with Quantile normalization was used to define differential gene expression fold changes in Biotique XRAY software. Genes having a minimum of 2 probesets with each probeset comprising of a minimum of 2 probes per probeset and found as present above background level were selected. Genes having differential expression with p<0.05 were considered. The analysis was done at full, extended and core probe levels.

The differential gene expression from exon array was also derived at core probe level using the ArrayTrack software. The gene expression at core probe levels was obtained from the Expression Console. In ArrayTrack software T test was performed with false detection rate (FDR) of 0.05 using the Benjamini and Hochberg approach, Mean Channel Intensity (MCI) of 100, spots flagged as bad spots (bad flag) 4. The gene annotations used for both analyses were from NCBI 36 mm8, Mus36 mRNA.bed, UCSC Mouse February 2006.

Analysis of differential gene expression in 3′IVT arrays was performed using Significance of Microarray Analysis (SAM) two class unpaired test [24] in the ArrayTrack (v 3.4.5) software with mean channel intensity of 100 and a false detection rate (FDR) of <0.05 and 0.001 using the Benjamini and Hochberg approach and bad flags 5. Fold change values with more than 2 were considered as significant for differential gene expression analysis.

To compare the Gene symbols, the double gene entries were removed. Genes having no confirmed Gene symbol till date from GenBank were removed; genes having no confirmed RefSeq id were removed.

## Results

We first compared data among different tissue groups to investigate the genetic changes occurring at different stages. The comparisons done were dysplasia versus transgenic tissue (D Vs Tn), dysplasia versus non-transgenic tissue (D Vs NTn), tumor versus transgenic tissue (Tm Vs Tn) and tumor versus non-transgenic tissue (Tm Vs NTn).

When the differentially expressed genes from 3′IVT array obtained from the ArrayTrack software were compared with differentially expressed genes from exon array as defined by the Biotique XRAY software we determined for 3′IVT arrays and at a FDR of <0.001 around 54 to 83% and for FDR of <0.05 around 52 to 70% similar gene regulations in the various group comparisons (Table 1 and 2, Files S1, S2, S3, S4, S5, S6, S7, S8, S9, S10, S11, and S12). To confirm that the change in direction of gene expression is the same for regulated genes we plotted scatter diagram of the fold change values obtained for these two types of arrays (Figures S1, S2, S3 and S4, panel A–C). While analyzing these comparisons we observed the direction of fold change is the same for genes found common in these comparisons except for few genes which are described in detail in the discussion section. Importantly, we found the fold change values for the genes common in both arrays and with common direction of change to be non-identical. The fold change values obtained from 3′IVT array were either lesser or more than that observed with whole transcript exon array and differences in the range of 0.5 times to 300 times were computed. When the type of probesets were considered for the calculation of gene expression changes from exon arrays, i.e. full, extended or core probesets the number of common genes and the expression values changed considerably (Table 1 and 2). When full probesets were taken into consideration the number of genes found to be expressed were increased so the numbers of common genes in the comparison were also increased. The number of differentially expressed genes followed the order core < extended < full in the various comparisons. At a fold change of ≥2 the number of differentially expressed genes was reduced for full probesets expressed genes. The Venn diagrams depicted in Figures 3–6 illustrate the overlap in the number of commonly regulated genes amongst the two platforms. At a FDR of <0.05 more common genes were found as compared with a FDR of <0.001. However, the percent of common genes was higher for a FDR of <0.001 as compared to FDR of <0.05 possible. Importantly, at a more stringent fixed FDR the confidence level for analysis for gene expression is increased. Here, at more stringent FDR most gene transcripts having proper GenBank accession number and RefSeq annotations were observed filtering out hypothetical gene transcripts which were observed at less stringent FDR. Thus, at a more stringent FDR less false positives were observed in the calculation of gene expression changes but this might be at the expense of more false negatives.

Next the fold change values of gene expression changes obtained from exon arrays considering core level probes using the ArrayTrack software were compared with fold change values of gene expression changes obtained for 3′IVT microarray. Here, data was solely analyzed with the ArrayTrack software. For exon arrays the FDR was set as 0.05 and mean channel intensity of 100 while for 3′IVT microarray the FDR was set as 0.05 and 0.001 and mean channel intensity of 100 with fold change above 2. The number of common genes found in these comparison for the different group comparisons were spanning over a broad range, i.e. 44% to 78% for a FDR of <0.001 and 15 to 64% for a FDR of <0.05 for 3′IVT arrays (Table 3 and 4, Files S13, S14, S15, S16 for FDR of <0.001 and Files S17, S18, S19 and S20 for FDR of <0.05). When a FDR of <0.05 was selected for 3′IVT array the direction of fold change was same for all genes in dysplasia versus transgenic and dysplasia versus non-transgenic tissues (Figure S1, S2, S3 and S4, panel D). However, when comparing tumor versus transgenic tissue gene *Kif26b* showed a change in direction and when comparing tumor versus non-transgenic tissue the genes *Mthfd1, Myom1* and *Ppp2r5c* equally differed in direction of gene expression. When a FDR of <0.001 was selected for 3′IVT microarray the change of direction for all genes in all groups was the same. With a FDR of <0.001 a more significant number of genes were selected and the direction of gene expression changes were the same filtering out the genes having opposite directions of fold change. Figure 7 depicts a Venn diagram to illustrate the overlap in gene regulation using data of 3′IVT and exon array at a fold change of ≥2.

## Discussion

Microarrays have matured as a robust technology in the determination of whole genome gene expression changes. Next to the 3′IVT array, RNA profiling at the exon level provides unprecedented opportunities for an identification of alternative splicing events in entire genomes. As of today only few studies attempted to cross-validate these two platforms. In the present study 3′IVT and exon array gene expression data was compared. We found direction of change, either increase or decrease, of gene expression changes to be similar for the genes found common in both arrays. However, the fold changes in gene expression in exon array compared to 3′IVT micro array are not equal. This may be explained by the method for calculation gene signal expression being different for both array types. The manufacture of exon arrays suggested that the gene expression signal obtained from exon array should not directly be used to compare with signals of 3′IVT array [25]. We therefore compared data on a fold change basis. The level of gene expression in exon array can be obtained by using full, extended or core probesets. While for some genes information for all 3 types of probesets are available, for others information may be based on extended and full probesets while some genes are detected only with full probesets depending on the gene sequence. Also the number of probesets per gene varies according to gene size and designated probe selection regions (PSR) for that gene [10], [26].

Previously, it has been shown that the gene expression in Affymetrix Human Exon Array (HuEx 1.0) has lower signal intensity as compared to Human 3′IVT array (U133 Plus 2) [11]. This report suggested the average responsiveness of probes on the exon array HuEx 1.0 is less than the probes of the 3′IVT array U133 Plus 2 as evidenced by dilution experiments with 25 spiked in transcript. In the present study fold change of differentially expressed genes between 3′IVT and exon array differed by 0.5 to 300 fold. Only few differentially expressed genes were identified when data of exon array was analyzed with highly stringent FDR of 0.001 using the ArrayTrack software, therefore we used less stringent FDR of 0.05 to identify significant differentially expressed genes. However, the criteria for exon array analysis using the Biotique XRAY were kept stringent to exclude false positive alternative splicing events that possibly affect an estimate of differentially expressed genes. Note, genes having less than 2 probesets assigned to mRNA were excluded. Some genes of the control, transgenic and non-transgenic groups may had minimal mRNA expression as compared to tumor or dysplasia tissue or likewise suppressed genes in tumor or dysplasia may had very low mRNA expression as compared to transgenic and non-transgenic tissue having expression near back ground level. These genes were also excluded.

A notable discrepancy in the gene expression fold change direction was observed when exon array data analyzed with Biotique XRAY software and 3′IVT array data analyzed with ArrayTrack software were compared. To investigate this further the exon transcript expression levels from these genes were analyzed in detail. We found some genes to undergo alternate splicing at the 3′ ends. These were *Trim37, Tanc2, Ptprd, Auts2, Tnfsf13b, Dysf, Epb4.1l5, Kif26b, Ppp2r5c, Rapgef5, Nxn* and *Rhobtb3*. In Table S1 the gene expression findings are summarized. For some genes there are more than 1 probesets in 3′IVT array and these were *Clic4, Eya1*, *Ifi205, Tsc22d1* and *Myh6*. Moreover, for few genes there are no transcripts with alternate 3′ end or no alternative probesets in 3′IVT array; these genes were *BC063749, Il4i1, A4galt* and *Gjb2*. Exon array provides summation of all exon transcripts to give gene expression levels while 3′IVT array consider only 3′ end. If transcripts with alternate 3′ end are present in different tissue groups due to alternative splicing, 3′IVT array might lead to wrong conclusions. Some of the genes which appeared to have opposite change of direction in exon array determined with the Biotique software showed similar direction change in gene expression as seen in 3′IVT when analyzed using the ArrayTrack software. These genes were *Tanc2, A4galt, Gjb2, Tnfsf13b, Nxn* and *Rhobtb3*.

Additional discrepancy in change of direction of gene expression was observed when tumor versus transgenic and tumor versus non-transgenic were analyzed using the ArrayTrack software for core level probesets of exon array. Here, data were compared with 3′IVT microarray gene expression fold change calculated at a FDR of <0.05. When comparing tumor versus transgenic tissue the gene *Kif26b* showed change in direction. This gene is known for different transcripts due to alternative splicing at the 3′end. By comparing tumor versus non-transgenic tissue the genes *Mthfd1, Myom1* and *Ppp2r5c* showed change in direction of gene expression. Note, the gene expression of *Mthfd1* was represented on the 3′IVT array and determined by probeset 1436704_x_at. This probe is considered to be the least significant probe of the mixed probeset for this gene. For the *Myom1* gene the untranslated mRNA sequence *AK052539* suggested by GenBank is given as alternatively spliced at the 3′end. Similarly, for *Ppp2r5c* different transcripts with alternative 3′ends are reported. When gene expression fold change for 3′IVT array was calculated at FDR of <0.001 and compared with results of the exon array the data was consisted without any controversy as the genes having change in direction of fold change were filtered out in the analysis.

For exon but not the 3′IVT arrays the use of the Biotique XRAY software is recommended [7]. However, the ArrayTrack software can analyze the data from both platforms at fold changes in gene expression [27]. Some microarrays like exon array can also be analyzed in ArrayTrack by manually uploading the Gene probe lists files obtained from the microarray manufacturer.

In conclusion, fold change gene expression changes can be used to compare data derived from exon and 3′IVT array but the fold changes are not the same. Using exon arrays has several advantages, notably to investigate gene expression changes of 3′ alternative spliced genes or to identify genes with either novel 3′ terminal exon or alternative polyadenylation sites or nonpolyadenylated messages and truncated transcripts. The fold change in transcript has to be confirmed with real time PCR to validate the data. T7 random primers used in exon array studies may be advantageous over oligo-dT primers used in 3′IVT array in situations like laser microdissected samples where RNA might be partly fragmented due to time consuming sample collection process and thus the poly A tail may not be as efficient in the amplification procedure in the laser micro-dissected samples. Finally, laser microdissected samples can be used for whole genome exon array analysis.

## Supporting Information

Figure S1 **Scatter plots for differentially expressed genes in the comparison dysplasia versus transgenic lung tissue.**
(TIF)

Figure S2 **Scatter plots for differentially expressed genes in the comparison dysplasia versus non-transgenic lung tissue.**
(TIF)

Figure S3 **Scatter plots for differentially expressed genes in the comparison tumor versus transgenic lung tissue.**
(TIF)

Figure S4 **Scatter plots for differentially expressed genes in the comparison tumor versus non-transgenic lung tissue.**
(TIF)

Table S1 **Gene expression comparison for the genes where change in direction of fold change due to alternate 3′ end.**
(DOC)

File S1 **List of genes found common in 3′IVT and exon array while comparing dysplasia vs transgenic groups.** 3′IVT array analyzed with ArrayTrack software with parameters as false detection rate (FDR) <0.001, fold change of ≥2, mean channel intensity (MCI) of 100, bad flag of 5. For exon array differential gene expression fold change obtained using Biotique XRAY software at core probeset level with p<0.05 using a minimum probeset per gene of 2 and a minimum of 2 probes per probeset.
(XLS)

File S2 **List of genes found common in 3′IVT and exon array while comparing dysplasia vs transgenic groups.** 3′IVT array analyzed with ArrayTrack software with parameters as false detection rate (FDR) <0.001, fold change of ≥2, mean channel intensity (MCI) of 100, bad flag of 5. For exon array differential gene expression fold change obtained using Biotique XRAY software at extended probeset level with p<0.05 using a minimum probeset per gene of 2 and a minimum of 2 probes per probeset.
(XLS)

File S3 **List of genes found common in 3′IVT and exon array while comparing dysplasia vs transgenic groups.** 3′IVT array analyzed with ArrayTrack software with parameters as false detection rate (FDR) <0.001, fold change of ≥2, mean channel intensity (MCI) of 100, bad flag of 5. For exon array differential gene expression fold change obtained using Biotique XRAY software at full probeset level with p<0.05 using a minimum probeset per gene of 2 and a minimum of 2 probes per probeset.
(XLS)

File S4 **List of genes found common in 3′IVT and exon array while comparing dysplasia vs non-transgenic groups.** 3′IVT array analyzed with ArrayTrack software with parameters as false detection rate (FDR) <0.001, fold change of ≥2, mean channel intensity (MCI) of 100, bad flag of 5. For exon array differential gene expression fold change obtained using Biotique XRAY software at core probeset level with p<0.05 using a minimum probeset per gene of 2 and a minimum of 2 probes per probeset.
(XLS)

File S5 **List of genes found common in 3′IVT and exon array while comparing dysplasia vs non-transgenic groups.** 3′IVT array analyzed with ArrayTrack software with parameters as false detection rate (FDR) <0.001, fold change of ≥2, mean channel intensity (MCI) of 100, bad flag of 5. For exon array differential gene expression fold change obtained using Biotique XRAY software at extended probeset level with p<0.05 using a minimum probeset per gene of 2 and a minimum of 2 probes per probeset.
(XLS)

File S6 **List of genes found common in 3′IVT and exon array while comparing dysplasia vs non-transgenic groups.** 3′IVT array analyzed with ArrayTrack software with parameters as false detection rate (FDR) <0.001, fold change of ≥2, mean channel intensity (MCI) of 100, bad flag of 5. For exon array differential gene expression fold change obtained using Biotique XRAY software at full probeset level with p<0.05 using a minimum probeset per gene of 2 and a minimum of 2 probes per probeset.
(XLS)

File S7 **List of genes found common in 3′IVT and exon array while comparing tumor vs transgenic groups.** 3′IVT array analyzed with ArrayTrack software with parameters as false detection rate (FDR) <0.001, fold change of ≥2, mean channel intensity (MCI) of 100, bad flag of 5. For exon array differential gene expression fold change obtained using Biotique XRAY software at core probeset level with p<0.05 using a minimum probeset per gene of 2 and a minimum of 2 probes per probeset.
(XLS)

File S8 **List of genes found common in 3′IVT and exon array while comparing tumor vs transgenic groups.** 3′IVT array analyzed with ArrayTrack software with parameters as false detection rate (FDR) <0.001, fold change of ≥2, mean channel intensity (MCI) of 100, bad flag of 5. For exon array differential gene expression fold change obtained using Biotique XRAY software at extended probeset level with p<0.05 using a minimum probeset per gene of 2 and a minimum of 2 probes per probeset.
(XLS)

File S9 **List of genes found common in 3′IVT and exon array while comparing tumor vs transgenic groups.** 3′IVT array analyzed with ArrayTrack software with parameters as false detection rate (FDR) <0.001, fold change of ≥2, mean channel intensity (MCI) of 100, bad flag of 5. For exon array differential gene expression fold change obtained using Biotique XRAY software at full probeset level with p<0.05 using a minimum probeset per gene of 2 and a minimum of 2 probes per probeset.
(XLS)

File S10 **List of genes found common in 3′IVT and exon array while comparing tumor vs non-transgenic groups.** 3′IVT array analyzed with ArrayTrack software with parameters as false detection rate (FDR) <0.001, fold change of ≥2, mean channel intensity (MCI) of 100, bad flag of 5. For exon array differential gene expression fold change obtained using Biotique XRAY software at core probeset level with p<0.05 using a minimum probeset per gene of 2 and a minimum of 2 probes per probeset.
(XLS)

File S11 **List of genes found common in 3′IVT and exon array while comparing tumor vs non-transgenic groups.** 3′IVT array analyzed with ArrayTrack software with parameters as false detection rate (FDR) <0.001, fold change of ≥2, mean channel intensity (MCI) of 100, bad flag of 5. For exon array differential gene expression fold change obtained using Biotique XRAY software at extended probeset level with p<0.05 using a minimum probeset per gene of 2 and a minimum of 2 probes per probeset.
(XLS)

File S12 **List of genes found common in 3′IVT and exon array while comparing tumor vs non-transgenic groups.** 3′IVT array analyzed with ArrayTrack software with parameters as false detection rate (FDR) <0.001, fold change of ≥2, mean channel intensity (MCI) of 100, bad flag of 5. For exon array differential gene expression fold change obtained using Biotique XRAY software at full probeset level with p<0.05 using a minimum probeset per gene of 2 and a minimum of 2 probes per probeset.
(XLS)

File S13 **List of common genes found when same platform of ArrayTrack software used for comparison between 3′IVT and exon array for comparing groups dysplasia vs transgenic.** For 3′IVT array differential gene expression fold change obtained with parameters as false detection rate (FDR) of <0.001, fold change of ≥2, mean channel intensity (MCI) of 100, bad flag of 5. For exon array differential gene expression fold change obtained at core probeset level with parameters as false detection rate (FDR) of <0.05, mean channel intensity (MCI) of 100, bad flag of 4.
(XLS)

File S14 **List of common genes found when same platform of ArrayTrack software used for comparison between 3′IVT and exon array for comparing groups dysplasia vs non-transgenic.** For 3′IVT array differential gene expression fold change obtained with parameters as false detection rate (FDR) of <0.001, fold change of ≥2, mean channel intensity (MCI) of 100, bad flag of 5. For exon array differential gene expression fold change obtained at core probeset level with parameters as false detection rate (FDR) of <0.05, mean channel intensity (MCI) of 100, bad flag of 4.
(XLS)

File S15 **List of common genes found when same platform of ArrayTrack software used for comparison between 3′IVT and exon array for comparing groups tumor vs transgenic.** For 3′IVT array differential gene expression fold change obtained with parameters as false detection rate (FDR) of <0.001, fold change of ≥2, mean channel intensity (MCI) of 100, bad flag of 5. For exon array differential gene expression fold change obtained at core probeset level with parameters as false detection rate (FDR) of <0.05, mean channel intensity (MCI) of 100, bad flag of 4.
(XLS)

File S16 **List of common genes found when same platform of ArrayTrack software used for comparison between 3′IVT and exon array for comparing groups tumor vs non-transgenic.** For 3′IVT array differential gene expression fold change obtained with parameters as false detection rate (FDR) of <0.001, fold change of ≥2, mean channel intensity (MCI) of 100, bad flag of 5. For exon array differential gene expression fold change obtained at core probeset level with parameters as false detection rate (FDR) of <0.05, mean channel intensity (MCI) of 100, bad flag of 4.
(XLS)

File S17 **List of common genes found when same platform of ArrayTrack software used for comparison between 3′IVT and exon array for comparing groups dysplasia vs transgenic.** For 3′IVT array differential gene expression fold change obtained with parameters as false detection rate (FDR) of <0.05, fold change of ≥2, mean channel intensity (MCI) of 100, bad flag of 5. For exon array differential gene expression fold change obtained at core probeset level with parameters as false detection rate (FDR) of <0.05, mean channel intensity (MCI) of 100, bad flag of 4.
(XLS)

File S18 **List of common genes found when same platform of ArrayTrack software used for comparison between 3′IVT and exon array for comparing groups dysplasia vs non-transgenic.** For 3′IVT array differential gene expression fold change obtained with parameters as false detection rate (FDR) of <0.05, fold change of ≥2, mean channel intensity (MCI) of 100, bad flag of 5. For exon array differential gene expression fold change obtained at core probeset level with parameters as false detection rate (FDR) of <0.05, mean channel intensity (MCI) of 100, bad flag of 4.
(XLS)

File S19 **List of common genes found when same platform of ArrayTrack software used for comparison between 3′IVT and exon array for comparing groups tumor vs transgenic.** For 3′IVT array differential gene expression fold change obtained with parameters as false detection rate (FDR) of <0.05, fold change of ≥2, mean channel intensity (MCI) of 100, bad flag of 5. For exon array differential gene expression fold change obtained at core probeset level with parameters as false detection rate (FDR) of <0.05, mean channel intensity (MCI) of 100, bad flag of 4.
(XLS)

File S20 **List of common genes found when same platform of ArrayTrack software used for comparison between 3′IVT and exon array for comparing groups tumor vs non-transgenic.** For 3′IVT array differential gene expression fold change obtained with parameters as false detection rate (FDR) of <0.05, fold change of ≥2, mean channel intensity (MCI) of 100, bad flag of 5. For exon array differential gene expression fold change obtained at core probeset level with parameters as false detection rate (FDR) of <0.05, mean channel intensity (MCI) of 100, bad flag of 4.
(XLS)

## References

[1] Tan PK, Downey TJ, Spitznagel EL, Xu P, Fu D (2003). Evaluation of gene expression measurements from commercial microarray platforms.. Nucleic Acids Res.

[2] Miklos GL, Maleszka R (2004). Microarray reality checks in the context of a complex disease.. Nat Biotechnol.

[3] Frantz S (2005). An array of problems.. Nat Rev Drug Discov.

[4] Abdullah-Sayani A, Bueno-de-Mesquita JM, van de Vijver MJ (2006). Technology Insight: tuning into the genetic orchestra using microarrays–limitations of DNA microarrays in clinical practice.. Nat Clin Pract Oncol.

[5] Ein-Dor L, Zuk O, Domany E (2006). Thousands of samples are needed to generate a robust gene list for predicting outcome in cancer.. Proc Natl Acad Sci U S A.

[6] Shi L, Reid LH, Jones WD, Shippy R, Warrington JA (2006). The MicroArray Quality Control (MAQC) project shows inter- and intraplatform reproducibility of gene expression measurements.. Nat Biotechnol.

[7] Affymetrix (2007). Application Focus: Whole-transcript Expression Analysis.. http://media.affymetrix.com/support/technical/appnotes/wt_appnote.pdf.

[8] Affymetrix (2005). GeneChip® Exon Array Design.. http://media.affymetrix.com/support/technical/technotes/exon_array_design_technote.pdf.

[9] Affymetrix (2004). GeneChip® Mouse Genome Arrays.. http://media.affymetrix.com/support/technical/datasheets/mogarrays_datasheet.pdf.

[10] Affymetrix (2005). Gene Signal Estimates from Exon Arrays - White paper.. http://media.affymetrix.com/support/technical/whitepapers/exon_gene_signal_estimate_whitepaper.pdf.

[11] Abdueva D, Wing MR, Schaub B, Triche TJ (2007). Experimental comparison and evaluation of the Affymetrix exon and U133Plus2 GeneChip arrays.. PLoS ONE.

[12] Zhang X, Liu G, Lenburg ME, Spira A (2007). Comparison of smoking-induced gene expression on Affymetrix Exon and 3′-based expression arrays.. Genome Inform.

[13] Gardina PJ, Clark TA, Shimada B, Staples MK, Yang Q (2006). Alternative splicing and differential gene expression in colon cancer detected by a whole genome exon array.. BMC genomics.

[14] Okoniewski MJ, Hey Y, Pepper SD, Miller CJ (2007). High correspondence between Affymetrix exon and standard expression arrays.. Biotechniques.

[15] Robinson MD, Speed TP (2007). A comparison of Affymetrix gene expression arrays.. BMC Bioinformatics.

[16] Rohrbeck A, Borlak J (2009). Cancer Genomics Identifies Regulatory Gene Networks Associated with the Transition from Dysplasia to Advanced Lung Adenocarcinomas Induced by c-Raf-1.. PLoS ONE.

[17] Rohrbeck A, Mueller VS, Borlak J (2009). Molecular Characterization of Lung Dysplasia Induced by c-Raf-1.. PLoS ONE.

[18] Kumar V, Abbas AK, Fausto N, Mitchell R (2007). Robbins Basic Pathology: Saunders..

[19] Kerkhoff E, Fedorov LM, Siefken R, Walter AO, Papadopoulos T (2000). Lung-targeted Expression of the c-Raf-1 Kinase in Transgenic Mice Exposes a Novel Oncogenic Character of the Wild-Type Protein.. Cell Growth Differentiation.

[20] Niyaz Y, Sägmüller B (2005). Non-contact laser microdissection and pressure catapulting: Automation via object-oriented image processing.. Medical Laser Application.

[21] Niyaz Y, Schütze K, Hayat MA (2005). 6 Noncontact laser microdissection and pressure catapulting: A basic tool in genomics, transcriptomics, and proteomics..

[22] Niyaz Y, Stich M, Sagmuller B, Burgemeister R, Friedemann G (2005). Noncontact laser microdissection and pressure catapulting: sample preparation for genomic, transcriptomic, and proteomic analysis.. Methods Mol Med.

[23] Schutze K, Niyaz Y, Stich M, Buchstaller A (2007). Noncontact laser microdissection and catapulting for pure sample capture.. Methods Cell Biol.

[24] Tusher VG, Tibshirani R, Chu G (2001). Significance analysis of microarrays applied to the ionizing radiation response.. Proc Natl Acad Sci U S A.

[25] Affymetrix Exon Array Analysis - Frequently asked questions_5. Affymetrix.. http://www.affymetrix.com/support/help/faqs/exon_array_analysis/faq_5.jsp.

[26] Affymetrix Exon Array Analysis - Frequently asked questions_9. Affymetrix.. http://www.affymetrix.com/support/help/faqs/exon_array_analysis/faq_9.jsp.

[27] Tong W, Cao X, Harris S, Sun H, Fang H (2003). ArrayTrack–supporting toxicogenomic research at the U.S. Food and Drug Administration National Center for Toxicological Research.. Environ Health Perspect.

