# Correction: A Cross-Platform Comparison of Genome-Wide Expression Changes of Laser Microdissected Lung Tissue of C-Raf Transgenic Mice Using 3′IVT and Exon Array

**DOI:** 10.1371/annotation/57bb672d-dd2f-4754-90ca-8575c1fd8903

**Published:** 2012-10-11

**Authors:** Kishor Bapu Londhe, Juergen Borlak

The following information is missing from the funding section: The charge for this publication was covered by the German Research Foundation (DFG) sponsorship "open access publication". 

