# Supplementary figures and images for: A Cross-Platform Comparison of Genome-Wide Expression Changes of Laser Microdissected Lung Tissue of C-Raf Transgenic Mice Using 3′IVT and Exon Array
Source: PLoS One. 2012 Jul 16;7(7):e40778. doi: 10.1371/journal.pone.0040778 (PMC3397940; doi:10.1371/journal.pone.0040778)

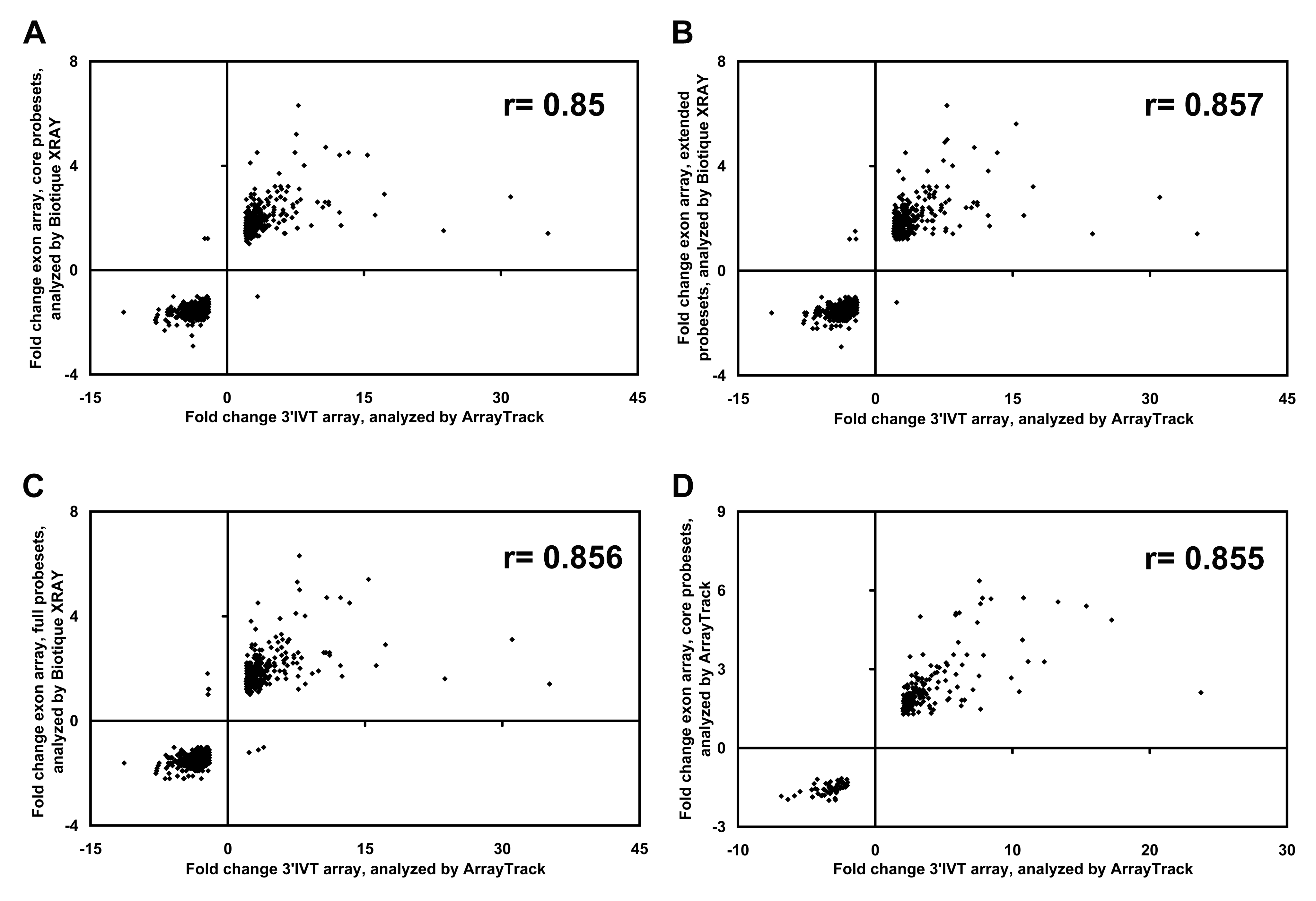

Supplement: Figure S1 — Scatter plots for differentially expressed genes in the comparison dysplasia versus transgenic lung tissue. (TIF) [file pone.0040778.s001.tif]

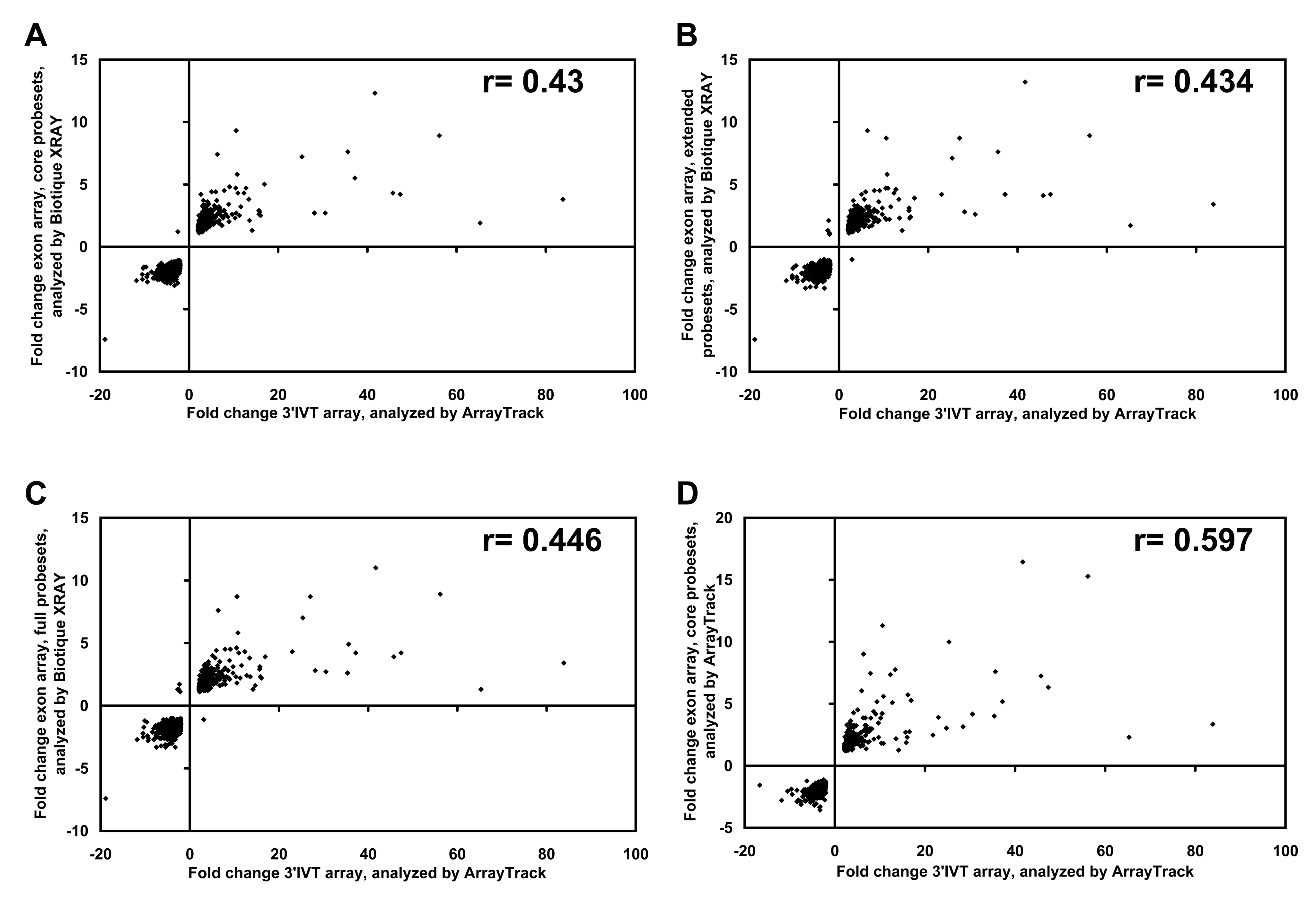

Supplement: Figure S2 — Scatter plots for differentially expressed genes in the comparison dysplasia versus non-transgenic lung tissue. (TIF) [file pone.0040778.s002.tif]

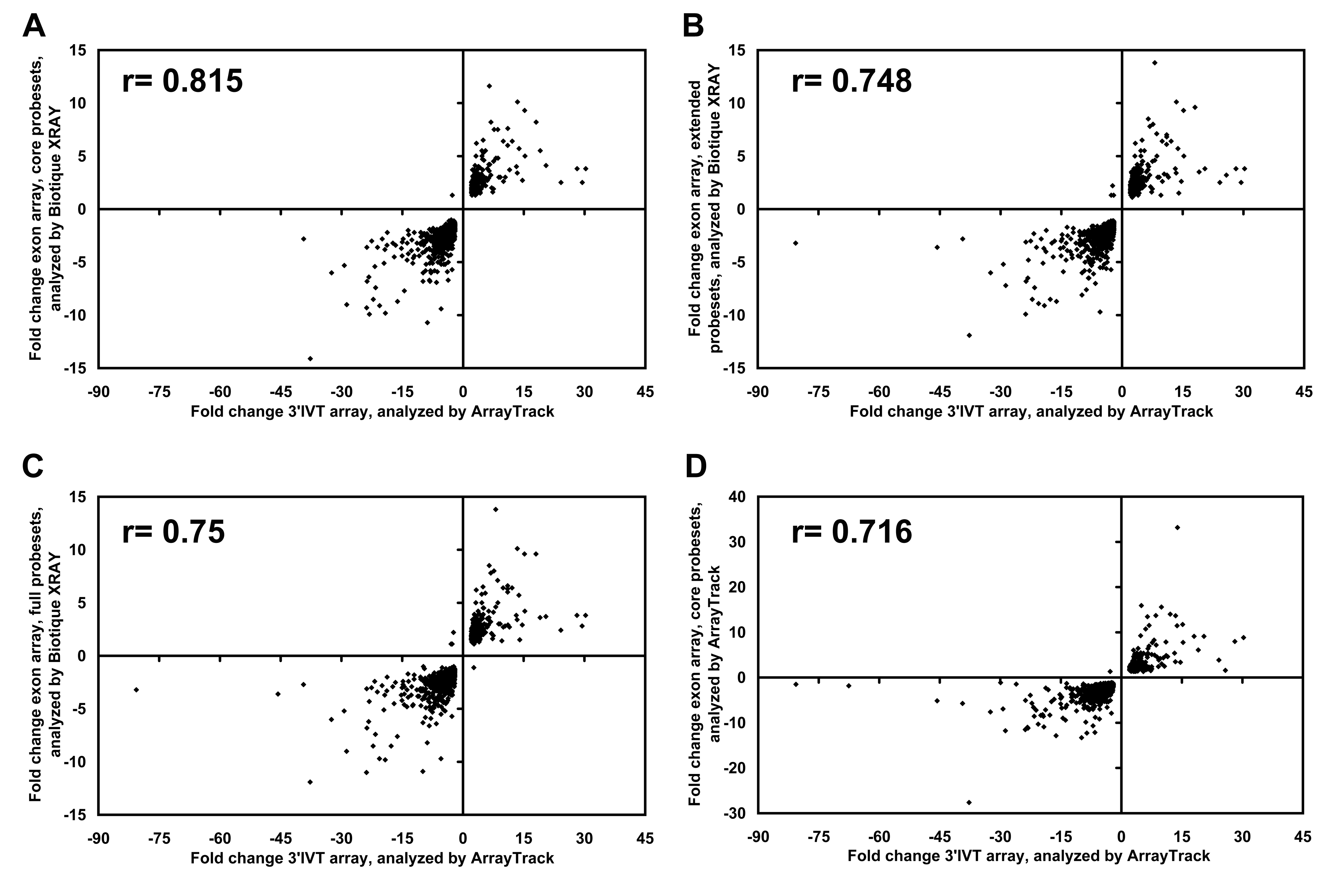

Supplement: Figure S3 — Scatter plots for differentially expressed genes in the comparison tumor versus transgenic lung tissue. (TIF) [file pone.0040778.s003.tif]

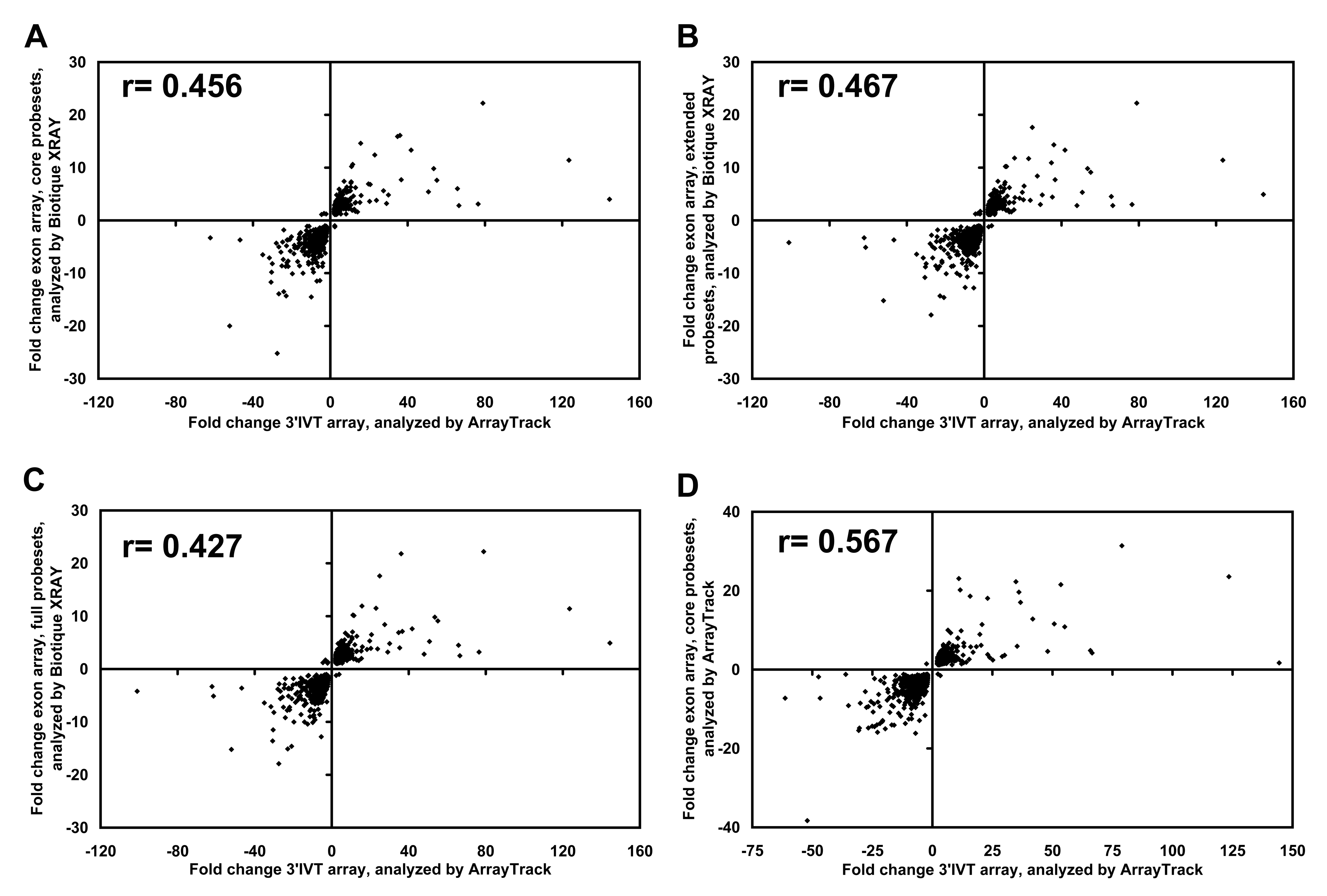

Supplement: Figure S4 — Scatter plots for differentially expressed genes in the comparison tumor versus non-transgenic lung tissue. (TIF) [file pone.0040778.s004.tif]
