# Supplementary material for: A Cross-Platform Comparison of Genome-Wide Expression Changes of Laser Microdissected Lung Tissue of C-Raf Transgenic Mice Using 3′IVT and Exon Array
Source: PLoS One. 2012 Jul 16;7(7):e40778. doi: 10.1371/journal.pone.0040778 (PMC3397940; doi:10.1371/journal.pone.0040778)
Supplement: Table S1 — Gene expression comparison for the genes where change in direction of fold change due to alternate 3′ end. (DOC) [file pone.0040778.s005.doc]

Supplementary table 1

Gene expression comparison for the genes where change in direction of fold change due to alternate 3’ end.

| Gene name | Expression in 3'IVT | Expression in exon array |
| --- | --- | --- |
| *Trim37* | -2.50 | 1.31 |
| *Tanc2* | 2.93 | -1.01 |
| *Ptprd* | -6.61 | 1.71 |
| *Auts2* | 3.12 | -1.11 |
| *Tnfsf13b* | -2.07 | 1.21 |
| *Dysf* | 2.33 | -1.21 |
| *Epb4.1l5* | -4.53 | 1.11 |
| *Kif26b* | -3.27 | 1.31 |
| *Ppp2r5c* | 2.29 | -1.01 |
| *Rapgef5* | -2.00 | 1.71 |
| *Nxn* | -2.19 | 1.21 |
| *Rhobtb3* | -2.32 | 2.21 |

Shown is the fold change in gene expression as determined by the ArrayTrack and Biotique XRAY software, respectively.
